# Knowledge, attitude and practices on cholera in an arid county, Kenya, 2018: A mixed-methods approach

**DOI:** 10.1371/journal.pone.0229437

**Published:** 2020-02-26

**Authors:** Erick Otieno Orimbo, Elvis Oyugi, Diba Dulacha, Mark Obonyo, Abubakar Hussein, Jane Githuku, Maurice Owiny, Zeinab Gura

**Affiliations:** 1 Field Epidemiology and Laboratory Training (FELTP) Kenya, Ministry of Health, Nairobi, Kenya; 2 County Government of Migori, Migori, Kenya; 3 Department of Health, County Government of Isiolo, Isiolo, Kenya; Chinese Academy of Medical Sciences and Peking Union Medical College, CHINA

## Abstract

**Background:**

Cholera remains a public health problem in Kenya despite increased efforts to create awareness. Assessment of knowledge, attitude and practice (KAP) in the community is essential for the planning and implementation of preventive measures. We assessed cholera KAP in a community in Isiolo County, Kenya.

**Methods:**

This cross-sectional study involved a mixed-methods approach utilizing a questionnaire survey and focus group discussions (FGDs). Using multistage sampling with household as the secondary sampling unit, interviewers administered structured questionnaires to one respondent aged ≥18 years old per household. We created knowledge score by allotting one point for each correct response, considered any total score ≥ median score as high knowledge score, calculated descriptive statistics and used multivariate logistic regression to examine factors associated with high knowledge score. In FGDs, we randomly selected the participants aged ≥18 years and had lived in Isiolo for >1 year, conducted the FGDs using an interview guide and used content analysis to identify salient emerging themes.

**Results:**

We interviewed 428 participants (median age = 30 years; Q1 = 25, Q3 = 38) comprising 372 (86.9%) females. Of the 425/428 (99.3%) who had heard about cholera, 311/425 (73.2%) knew that it is communicable. Although 273/428 (63.8%) respondents knew the importance of treating drinking water, only 216/421 (51.3%) treated drinking water. Those with good defecation practice were 209/428 (48.8%). Respondents with high knowledge score were 227/428 (53.0%). Positive attitude (aOR = 2.88, 95% C.I = 1.34–6.20), treating drinking water (aOR = 2.21, 95% C.I = 1.47–3.33), age <36 years (aOR = 1.75, 95% C.I = 1.11–2.74) and formal education (aOR = 1.71, 95% C.I = 1.08–2.68) were independently associated with high knowledge score. FGDs showed poor latrine coverage, inadequate water treatment and socio-cultural beliefs as barriers to cholera prevention and control.

**Conclusions:**

There was a high knowledge score on cholera with gaps in preventive practices. We recommend targeted health education to the old and uneducated persons and general strengthening of health education in the community.

## Introduction

Cholera is an acute disease caused by the bacterium *Vibrio cholerae* and is characterized mainly by watery diarrhea. [1,2]. The bacterium has more than 200 serogroups but only serogroup O1 and O139 which thrive in crowded housing conditions with poor sanitation have been associated with outbreaks worldwide. [3]. They produce the cholera toxin (CT) which is responsible for most of the clinical manifestations of the disease [4]. Confirmatory diagnosis of cholera is by isolation of organism in stool via culture [5] and rapid tests are used for provisional diagnosis. Administration of fluids and electrolytes is the mainstay of treatment and antibiotics are administered in case of severe dehydration. Proper control requires a combination of public health surveillance, water sanitation and hygiene, social mobilization, treatment and vaccination using oral cholera vaccine [2].

Cholera is a global public health problem and an indicator of social inequity affecting 1.3–4 million people annually with about 21,000–143,000 deaths globally [6]. About 2.8 million cases and 91,000 deaths occur in Africa annually [3,6]. In Kenya, 15 discrete outbreaks had been identified from 1971–2010. In 2008, about 790 cases and 53 deaths (CFR 6.7%) were reported in an outbreak in western Kenya [7]. Another major outbreak between January 2009 and May 2010 resulted in 274 deaths amongst 11,769 cases (CFR 2.3%) in 52 districts (sub-counties) throughout the country [8]. There have been outbreaks in various counties between December 2015 and January 2018 with 21,066 cases and 325 deaths (CFR 1.5%) reported [9]. Between January 1, 2017 and November 29, 2017 about 20/47 (43%) of the counties in Kenya had reported 3,967 cases including 76 deaths (case fatality rate = 1.9%) with 596 cases reported being laboratory confirmed [10].

Isiolo County is located in the arid upper eastern Kenya. The had reported several cholera outbreaks between 2017 and 2018. Individual and community knowledge, behaviors, attitudes and practices related to sanitation have been reported to have a direct impact on the prevention, control and management of cholera. This study assessed the community knowledge, attitude and practices in relation to cholera in Isiolo County in February 2018.

## Materials and methods

### Study setting

Isiolo County is divided into three sub-counties namely Isiolo, Merti and Garbatulla (Isiolo South) (S1 Fig) and covers an area of approximately 25,700 km^2^. In 2017, the human population was estimated at 191,627 with about 36,953 households. [11]. The climate is mainly hot and dry with long rains from March to May, and short rains from October to November. The population consists largely of nomadic pastoralists who live in rural areas where access to safe drinking water is limited; 58% of the water sources have saline water [12]. Spatially, 93% of the residents lack access to safe drinking water within a five-kilometer radius. Water sources that are operational during the wet season are 59% and those operating in the dry season are 36%. About 81% of the households have pit latrines of which 56% are uncovered. Households that are connected to the sewerage line are roughly six percent and are found in Isiolo Town [12].

### Study design

We did a cross-sectional study utilizing a mixed-methods approach that involved community knowledge, attitude and practices (KAP) survey and focus group discussions (FGDs). Different participants were enrolled for the two activities such that no individual took part in both activities. We conducted three FGD; two had only female participants and one had only male participants to accommodate the community’s cultural sensitivities about mixing women and men in meetings.

### Study participants

Trained interviewers administered questionnaires to female heads of households who are traditionally regarded as the main decision-makers in matters of food preparation and water sanitation and hygiene [13]. In the absence of the female head of a household, the next senior most member of the household aged ≥18 years was interviewed. The interviewers included all the households where the respondents were present. Interviewers moved to the next available household in the event the selected one had no respondent meeting the inclusion and consent criteria. To participate in the FGDs, a community member should have been aged ≥18 years and should have been a resident of Isiolo County for a duration of at least one year.

### Sample size assumptions and calculation

Cochran’s formula for estimating the differences in proportions was used to calculate sample size [14]. We assumed a prevalence of 84% based on the proportion of respondents which had high knowledge according to a past Kenyan study [15], the precision of study of 0.05 at 95% confidence level and applied a design effect of two to correct for variability in the clusters within the community. We obtained a minimum sample size of 412 households for the community KAP survey.

### Sampling strategy

A multistage sampling approach was used and the household was the secondary sampling unit. We randomly selected Isiolo Sub-County out of the three sub-counties and included all the wards in the sub-county namely Bulapesa, Wabera, Burat, Oldonyiro and Ngaremara. We used probability proportionate to size sampling to allocate the sample size to all the five wards based on the number of the households per ward. Within the wards, trained interviewers sought and obtained a list of all health facilities—community dispensaries, health centers or any public health facility. These were used as central reference points. The sample size was equally allocated among the central reference points in each ward. They then randomly picked the first household by spinning a 500ml water bottle and using the direction of the bottle top to determine the direction in which to start sampling. They subsequently selected the households systematically (every fifth household in the pointed direction) until they achieved half the sample size. The interviewers then went back to the central reference point and moved in the opposite direction for the remaining half.

We conducted gender-specific FGDs by randomly sampling, selecting and inviting the participants with the help of the local community health volunteers (CHVs) who approached them face-to-face. The participants were unfamiliar with the investigators; however, some were familiar with the CHVs. The CHVs invited eligible community members from the study area for a meeting in a central place (local health facility). They were approached at their households. All the members who came were issued with papers with unique numbers. We used a random number generator in Microsoft Excel software to randomly pick the numbers after considering the total number of participants required for each FGD. We selected participants whose unique numbers were picked and there were no refusals.

### Data collection

Using an electronic questionnaire in portable data assistants (PDAs), trained interviewers collected information on socio-demographic factors, knowledge, attitude and practices on cholera among the community members. We used a set of 13 questions to assess knowledge on cause, transmission, treatment and prevention of cholera and another set of nine questions to assess water hygiene and sanitation practices. Attitude was assessed by asking how the respondents' perceived cholera compared to other causes of diarrhea in terms of severity. We instructed the interviewers to observe for the presence of toilets/latrines, water treatment chemicals and household soap. We considered their observation of these as an indicator of the use of toilets/latrines, water treatment and washing of hands with soap. The respondents were asked the question and the same time informed that the interviewer is interested in making observations.

The FGDs were conducted in the local Borana language complemented with Swahili using an interview guide. The guide was both in Borana and Swahili languages. The local CHVs provided the translations from Borana to Kiswahili and vice-versa. Each FGD lasted about 60 minutes. The FGDs were held in the halls within the local health facilities and non-participants were absent in the halls. Two investigators moderated the discussions and probed the responses. We asked open-ended questions to elicit information on knowledge, attitudes, practices, health-seeking behavior and socio-cultural practices related to cholera. We asked a concluding question on what the participants would say were the most important issues they wanted to express regarding prevention of cholera and other diarrheal diseases. Data were captured through note-taking and audio recording. We used the notes and the audio recordings to verify each script and later transcribed them into written form word for word.

### Data analysis

Data were cleaned and analyzed using Epi-Info version 7 (CDC, Atlanta, GA). The responses on knowledge were scored as one for a correct response and zero for an incorrect response or *“I don’t know”* response. The scores were added to obtain a total score for each respondent and a median score was calculated. High knowledge score was defined as a total score above or equal to the median score and low knowledge score as a score below the median score. Those who perceived cholera as more severe than other common diarrheal diseases were classified as having a positive attitude and those who perceived cholera as less or equally severe as having a negative attitude. Good defecation practice was defined as having a flush toilet or covered pit latrine and poor defaecation practice as having uncovered pit latrines, flying toilets or practicing open defecation. Formal education was defined as having attained basic schooling (Koranic, primary, secondary or tertiary education). Respondents who had not attended any school were considered to have had no formal education.

We described the socio-demographic characteristics, calculated means, medians and corresponding measures of dispersion for continuous variables (median for variables with skewed number distributions {with outliers} and means for variables with normal number distributions) and proportions for categorical variables. Using high knowledge score as the dependent variable in the bivariate analysis after we had adjusted for the clustering of the households in the wards, we calculated crude odds ratios (cOR) and their corresponding 95% confidence intervals; variables with p-values <0.1 were included in a logistic regression model to obtain the adjusted odds ratios (aOR). Variables with p-value <0.05 in the regression model were considered to be statistically significant. We used a backward stepwise elimination method to identify factors independently associated with high knowledge score.

We translated the qualitative information collected from the FGDs transcripts and analyzed the data using content analysis method. This process used a sequence of steps from reading, coding, displaying, reduction and interpretation. We carefully read the transcripts, coded the data, refined codes accordingly and identified salient emerging themes during data interpretation. Two investigators coded the data. We presented the findings in prose and used direct quotations from the translated transcripts to present some of our findings.

### Ethical considerations

Isiolo County Department of Health gave authorization of the study and the interviewers obtained informed consent from all the study participants. The consent was verbal. The study did not include minors. Kenya’s Ministry of Health through Kenya Field Epidemiology and Laboratory Training Program approved the study. We de-identified and stored all responses from the participants in password-protected computers. The FGD participants were assured that the discussions were anonymous despite audiotaping and the audiotapes were safely stored in a password-protected computer.

## Results

### Descriptive findings

#### Sociodemographic characteristics for community KAP respondents

The team visited 428 households and interviewed 428 individuals. The median age of all participants was 30 years (first quartile = 25, third quartile = 38), mean household size was 5.7 people (standard deviation = 5.4) and mean number of children under five years was 1.1 (standard deviation = 1.4) (Table 1).

**Table 1:** Sociodemographic characteristics of the respondents of community KAP on cholera, Isiolo County, February 2018.

Variable	Frequency	Percent
**Wards of residence (n = 428)**		
Bula Pesa	145	33.9
Wabera	93	21.7
Burat	87	20.3
Oldonyiro	77	18.0
Ngaremara	26	6.1
**Sex (n = 428)**		
Female	372	86.9
Male	56	13.1
**Age group (n = 425)**		
18–35	291	68.5
36–49	91	21.4
50+	43	43.1
**Level of education (n = 421)**		
Primary Completed (8 years)	125	29.7
No formal education	132	31.3
Secondary completed (12 years)	63	15.0
Secondary incomplete (<12 years)	49	11.6
Tertiary (>12 years)	27	6.4
Primary incomplete (< 8 years)	25	5.9
**Source of family income (n = 413)**		
Small-scale business	172	41.7
Unskilled labor	105	25.5
Unemployed	69	16.7
Formal employment	55	13.2

*KAP- Knowledge, Attitudes and Practices

#### Sociodemographic characteristics for community FGDs participants

The three FGDs had 10 participants each in Wabera (FGD I), Bulla pesa & Burat (FGD II) and Ngaremara (FGD III) wards of Isiolo Sub-County. The FGD I had only men while FGD II and III had only women. The 30 FGD participants had a mean age of 34.2 years (standard deviation = 12.3 years). Amongst the participants, 11 (36.7%) had achieved upper primary school education (8 years), 9 (30%) did not have any formal education, seven (23.3%) achieved secondary school education (12 years) and three (10%) had tertiary education (more than 12 years). There were 20 (66.7%) women, 19 (63.3%) Muslims and 11 (36.7%) Christians.

#### Knowledge assessment

On knowledge assessment, 425/428 (99.3%) had heard of cholera, 311/425 (73.2%) knew cholera can spread within the community and 325/428 (75.9%) mentioned poor hygiene and sanitation as a main predisposing factor. Cholera symptoms reported were watery diarrhea 384 (89.7%) and vomiting 321 (75.0%). Those who knew washing hands and treating drinking water were important prevention measures were 317/428 (74.1%) and 273/428 (63.8%) respectively (Table 2).

**Table 2:** Community knowledge on cholera, Isiolo County, February 2018 (n = 428).

Causes of cholera	Frequency	Percent
Poor hygiene	325	75.9
Drinking contaminated water	245	57.2
Eating contaminated food	137	32.0
Open defecation	109	25.5
Unwashed fruit/vegetables	62	14.5
Flies/insects	55	12.9
Don’t know	14	3.3
Spirits/curse/ bad omen	3	0.7
**Symptoms of cholera**		
Watery diarrhea	384	89.7
Vomiting	321	75.0
Abdominal pain	47	11.0
Dehydration	38	8.9
Fever	18	4.2
Bloody diarrhea	14	3.3
Malaise/lethargy	12	2.8
Joint pain	6	3.7
Nausea and Decreased appetite	6	1.4
**Ways of preventing cholera**		
Wash hands	317	74.1
Boil or treat water	273	63.8
Use a latrine/ avoid open defecation	151	35.3
Cook food thoroughly	141	32.9
Clean cooking utensils/vessels	140	32.7
Wash vegetables and fruit	78	18.2
Cover food	68	15
Reheat stored food	29	6.9
Cannot prevent	9	2.1

Additionally, 322/428 (75.2%) respondents had heard about oral rehydration salts (ORS) and 271/322 (84.2%) knew how to prepare it. Among them, 152/ 322 (47.2%) reported that ORS was available in their village and 225/322 (69.9%) had been taught how to prepare homemade ORS. Those who had heard about water treatment products were 283/421 (67.2%). The overall proportion of respondents with high knowledge score was 227/428 (53.0%).

For FGDs, majority of the participants mentioned poor hygiene, open defecation, drinking untreated water, flies, eating unwashed fruits and vegetables and the dirty environment as predisposing factors for cholera outbreaks. Signs and symptoms elicited were; any continuous diarrhea, watery diarrhea up to 3 episodes or more than 4 episodes, rice watery diarrhea, vomiting and diarrhea, stomach cramps, malaise, paleness and dehydration. The main theme on prevention from the participants was maintaining a high standard of hygiene including drinking of treated water, washing hands before cooking, eating and after visiting the toilets and use of toilets and latrines as captured in the excerpt below:

“*We are supposed to treat our drinking water*, *wash our hands before cooking*, *eating and after visiting the latrine/toilet*. *We are always supposed to use latrine/toilets*. *Generally*, *it is about maintaining cleanliness*.*”*

Most participants mentioned giving water with salt and sugar as a home treatment for cholera. Other responses included giving ORS solution if available at home and increased fluid intake (water, soup, any fluid) and giving herbs. A participant said, “*We give traditional alcoholic beverage mixed with herbs*.*”* Most participants said they would report any suspected cholera to the nearest health facility and mentioned doxycycline, which they referred to as “*green capsules*”, as a drug used to treat cholera.

#### Attitude assessment

Respondents who perceived cholera as more severe than other diarrhoeal diseases were 383/425 (90.1%).

For FGDs, most participants said cholera is more severe than other diarrheal diseases. A participant said that cholera is associated with stigma as documented in the excerpt below:

“*Cholera case-patients are perceived as dirty or unable to maintain hygiene in their households*.*”*

Poor environmental hygiene was reported as the main reason for the increase in diarrheal illnesses in Isiolo especially open defaecation, general lack of awareness on handwashing, unregulated roadside eateries and shallow ponds that were likely to be contaminated. Some participants mentioned the drinking of untreated water. Other participants also attributed cholera cases to supernatural powers; a participant said, *“It is due to God’s will*.*”*

The participants also mentioned socio-cultural beliefs that might lead to the spread of cholera:

“*Local community do not consider infants*’ *feces as harmful*.”

“*Nomadic-pastoralism is incompatible with good hygiene making cholera prevention difficult*.*”*

“*I have never used any of the water treatment products but my family and I are healthy despite using untreated water*. *This is because of God’s will*.*”*

Most said they would be interested in cholera prevention because treatment is expensive. Most participants mentioned some of the water treatment chemicals that were readily available in shops and dispensaries. They felt that these water treatment agents were not readily accepted by the pastoralists and had an unpleasant smell and taste when put in drinking water. Some participants preferred boiling water to using water treatment products to make water safe for drinking and a few of the participants felt the products were important as they assisted in making water safe:

“*The medicines are expensive*.*”*

“*We have water treatment tablets in the local shops*, *markets and in the health facilities but most of our people don’t use them because of unpleasant smell and taste*.*”*

“*We pastoralists consider drinking water very precious*, *we don’t like adulterating it before drinking*.*”*

#### Practices assessment

In the KAP survey, respondents who had good defecation practice were 209/428 (48.8%): 183/428 (42.8%) reported using covered pit latrines and 26/428 (6.1%) used flush toilets. Those who used uncovered pit latrines were 176/428 (41.1%), 23/428 (5.4%) defecated in the bushes and 16/428 (3.7%) used their neighbors’ latrine. Water was not available for over 3 months in a year for 348 (81.3%) respondents and treatment of water before drinking was practiced by 216/421 (51.3%) respondents. Those who had soap in their households were 352/428 (82.2%) and 323/428 (75.5%) reported always washing their hands using soap. Handwashing after visiting the toilet was practiced by 383/428 (89.5%) of respondents, handwashing before eating by 362/428 (84.6%) and handwashing before cooking by 173/428 (40.4%) of the respondents (Table 3). Interviewer observations did not differ with responses from the participants where the indicator of practice was observed.

**Table 3:** Community practices on handwashing, Isiolo County, February 2018 (n = 428).

Practices on handwashing	Frequency	Percent
After visiting the toilet	383	89.5
Before eating	362	84.6
Before cooking	173	40.4
After changing babies napkin	88	20.6
When hands are visibly dirty	76	17.7
When serving meals	68	15.9
After cleaning babies when they defecate	48	11.2
Others	11	2.6

In the FGDs, most participants felt that there was general improvement in the level of environmental hygiene, sanitation and water hygiene in their wards the last few years; some reported that the proportion of the population using open defecation had reduced; few reported that the area experienced water shortages that hindered personal and environmental hygiene. A participant in one of the FGDs said, *“We are open defecation free”*. Participants reported the presence of monthly waste collection system by the county government and presence of piped water supply in their ward and that most households had latrines within Isiolo town. They felt this contributed to the general improvement in hygiene. In one FGD conducted in a rural area, the participants mentioned poor latrine coverage as one of the hindrances to environmental hygiene. They also highlighted drought as a factor that affected the availability of safe drinking water. In another FGD, all participants got water from the borehole; others treated this water while others did not. Most participants mentioned they defecate in communal latrines without cover. Few latrines are covered. Some households used open defaecation. Most participants reported washing hands after visiting the toilet/latrine, before eating, before cooking and after changing babies’ nappies. Two Muslim participants said it was a religious obligation to wash hands immediately after waking up and when preparing for prayers. Most participants in one FGD said they did not treat their water because they believed the water was fit for human consumption and they lacked the water treatment products. Two participants said they boiled the water. In another FGD, most participants who used tap water reported not treating it because the distribution company already treated it while some boiled the water. Three participants said they did not treat their water while the remaining treated using available chemicals.

### Factors associated with high knowledge score on cholera

In the bivariate analysis, those with high knowledge score on cholera had three times the odds of having a positive attitude compared to those with low knowledge score (cOR = 3.12, 95% C.I = 1.59–6.12). Those with high knowledge score on cholera had twice the odds of treating their drinking water compared to those with low knowledge score (cOR = 2.17, 95% C.I = 1.47–3.21). Those with high knowledge score on cholera had twice the odds of being aged below 36 years compared to those with low knowledge score (cOR = 2.17, 95% C.I = 1.43–3.30). Those with high knowledge score on cholera had twice the odds of having some formal education compared to those with low knowledge score (cOR = 1.96, 95% C.I = 1.28–3.00). Those with high knowledge score on cholera had almost thrice the odds of having soap in the house compared to those with low knowledge score (cOR = 2.63, 95% C.I = 0.73–0.80).

In the multivariate analysis, having a positive attitude (aOR = 2.88, 95% C.I = 1.34–6.20), treating drinking water (aOR = 2.21, 95% C.I = 1.47–3.33), being aged below 36 years (aOR = 1.75, 95% C.I = 1.11–2.74) and having some formal education (aOR = 1.71, 95% C.I = 1.08–2.68) were independently associated with having high knowledge score. Washing hands after visiting the toilet, sex and defecation practice were not entered into the multivariate model because they had a P-value of more than 0.1 at the bivariate level. (Table 4).

**Table 4:** Factors associated with high knowledge score on cholera amongst residents of Isiolo County, Kenya, February 2018.

Variables	N	High knowledge score n (%)	Low knowledge score n (%)	cOR ^a^	95% C.I ^b^	aOR ^c^	95% C.I
Treating water (n = 421)							
Yes	216	135 (60.3)	81 (41.1)	2.17	1.47–3.21	**2.21**	**1.47–3.33**
No	205	89 (39.7)	116 (58.9)	*Ref.			
Age Group in years (n = 425)							
<36	291	173 (76.2)	118 (59.6)	2.17	1.43–3.30	**1.75**	**1.11–2.74**
36+	134	54 (23.8)	80 (40.4)	*Ref.			
Attitude category (n = 428)							
Positive	383	214 (55.9)	169(84.1)	3.12	1.59–6.12	**2.88**	**1.34–6.20**
Negative	45	13 (28.9)	32 (15.9)	*Ref.			
Formal education (n = 421)							
Yes	297	174 (77.0)	123 (63.1)	1.96	1.28–3.00	**1.71**	**1.08–2.68**
No	124	52 (23.0)	72 (36.9)	*Ref.			
Presence of soap in the house (n = 364)							
Yes	352	200(98.0)	152 (95.0)	2.63	0.78–0.80	1.63	0.43–6.11
No	12	4 (2.0)	8 (5.0)	*Ref.			
Washing hands after visiting the toilet (n = 428)							
Yes	383	206 (90.8)	177 (88.1)	1.33	0.71–2.47		
No	45	21 (9.2)	24 (11.9)	*Ref.			
Sex (n = 428)							
Male	56	31(13.7)	25 (12.4)	1.11	0.63–1.96		
Female	372	196 (86.3)	176 (87.6)	*Ref.			
Defecation practice (n = 428)							
Good	209	112 (49.3)	97 (48.3)	1.04	0.71–1.53		
Poor	219	115 (50.7)	104 (51.7)	*Ref.			

NB-Washing hands after visiting the toilet, sex and defecation practice were not entered into the multivariate model because they had a P-value of more than 0.1 at the bivariate level

^a^ crude odds ratio

^b^ confidence intervals

^c^ adjusted odds ratio

*Ref.—reference group

## Discussion

Our study found that over half of the respondents had high knowledge score on cholera including its causes, modes of transmission and methods of preventing cholera transmission. Majority of the participants from both KAP survey and FGDs had a positive attitude and half of the respondents treated their water before drinking. Slightly over half of the respondents had good defecation practice (either had a flush toilet or used a covered pit latrine). High knowledge score did not necessarily translate into good practice and participants who reported knowing the importance of treating water were more than those who reported they practiced treatment of water before drinking.

A majority of respondents with high knowledge score knew about the importance of handwashing and reported practicing hand washing before eating and after defecation. Therefore, in this instance, high knowledge score translated into good practice. Regarding handwashing after defecation, our findings are similar to those of a study in Bangladesh that found majority (90%) of the respondents had high knowledge score about handwashing after defecation and 88% washed hands after defecation. In contrast, few (21%) respondents in the Bangladesh study reported handwashing before eating [16]. The Bangladeshi study was a cross-sectional comparative study between baseline, midline and end-line surveys while our study was a one-off cross-sectional survey and that could explain the difference in findings. However, other studies in Kenya have also documented the low proportion of persons who practice handwashing before eating or after defecation despite having high knowledge score [17,18]. Our study area could have benefitted from health education and advocacy on hand hygiene conducted during past cholera outbreaks hence the high proportion of respondents who washed hands after defecation and before eating.

Quite a number of the respondents perceived cholera to be more severe than other diarrhoeal diseases. We attribute this to the awareness that cholera can quickly progress to cause severe dehydration resulting in death if no immediate medical attention is given. Some respondents had experiences where they lost relatives to cholera while being rushed to a hospital and so the experience reinforced their belief that cholera is more severe compared to other diarrheal diseases. In the FGDs, we found cultural perceptions that may contribute to the spread of cholera in the community like the belief that a young child’s stool cannot be a source of cholera infection and so children can practice open defecation since their stool is harmless. A similar cultural belief that children’s stool is harmless and cannot transmit cholera was reported in a study conducted in Tanzania [19]. Such perceptions increase contamination of water sources by fecal matter and contribute to the spread of cholera during outbreaks. Our study area has water scarcity since it is majorly arid and semi-arid land (ASAL) and contamination of the few water sources could be contributing to the recurrent and sometimes protracted cholera outbreaks in the ASALs.

High knowledge score was also associated with respondent having a positive attitude leading to practices that contribute to prevention and control of diarrheal diseases. Respondents who had high knowledge score treated water before drinking. Treatment of water before drinking at the household level and storing drinking water in sanitary conditions is vital to prevent cholera outbreaks [20]. Knowledge also corrects the negative socio-cultural beliefs and ignorance that can lead to diarrheal disease outbreaks, for example, believing that children’s stool is harmless. Respondents who had attained some level of formal education had high knowledge score, possibly because education improves literacy and these respondents are able to understand health information, whether in the health facility or in the community. Our study area had community health volunteers who educate the community on hygiene and sanitation. This underscores the need to reach even the pastoralists who have no education, to ensure they also have the correct hygiene and sanitation practices. Another finding was that mainly those respondents who were less than 36 years old had high knowledge score on cholera. In pastoralist communities, younger people tend to have a better education than older people do: our study shows than high knowledge score is associated with education. We also postulate that exposure to other cultures and information through various channels like mass and social media by the respondents under 36 years old may have led to high knowledge score. Findings from a study in an area with a high incidence of cholera in Bangladesh also reported that high knowledge score on cholera was associated with positive attitude, practices and education [21].

Some differences in findings between the quantitative study and the FGDs was noted, for example, most FGD participants reported washing hands before cooking and after changing babies’ napkins but less than half of survey participants reported practicing such behavior. We postulate that these differences could be due to a larger sample size and respondents providing more truthful responses in the survey. In FGDs, there is some degree of group influence towards how people respond to questions, for example, overstating their actual practices. The difference could also be due to different data analysis methods.

We postulate that water scarcity during certain times of the year and failure to treat drinking water by about half the respondents is a major constraint in cholera prevention in Isiolo County; only half of the respondents treated their drinking water.

This study was not without limitations. Some respondents with high knowledge score on cholera may have not been truthful about their actual practices; it is possible that some respondents’ claims exceeded their actual practices on cholera prevention, which might have introduced response bias in the study. We instructed the interviewers to observe for the presence of toilets/latrines, water treatment chemicals and household soap as indicators of the use of toilets/latrines, water treatment and washing of hands with soap. By informing the respondents on this prior to asking questions, this potential bias was minimized as the responses did not differ from the observations. However, this bias could not be completely ruled out with other practices that could not be observed. We also complemented our community survey with three FGDs with different participants. This study was a cross-sectional survey hence might only measure knowledge, attitude and practice as at the time the study was conducted and cannot be used to analyse behaviour over time. The snapshot nature of the study design cannot also guarantee representativeness. Unavailability of respondents in the selected households might have also introduced an element of bias.

## Conclusions and recommendations

There was a high knowledge score about cause and transmission of cholera and measures that can be instituted to prevent cholera outbreaks in the community. However, some gaps were clear in various aspects of practices such as water treatment and defecation practices (use of either covered latrine or flush toilet). The community attitudes and perceptions of cholera were influenced by knowledge of the severity of the disease and socio-cultural beliefs. Some of these beliefs could lead to the spread of cholera in the community. Findings from the FGDs indicated preventive measures such as public awareness programs on cholera control and improvement of latrine coverage are necessary for the community. Barriers to cholera prevention and control, were low latrine coverage, inadequate water treatment and socio-cultural beliefs.

We recommend targeted health education to the old and uneducated in the community and regular health education in the community to improve knowledge and preventive practices such as water treatment and use of toilets and latrines. Another recommendation is for the County Government of Isiolo to improve the supply of clean and safe water in the community by sinking and desalinating boreholes to the recommended salinity levels. The County should also improve the coverage of covered pit latrines by use of strategies like provision of subsidies in the form of construction materials for pit latrines and advocacy to educate the community on dangers of open defecation.

## Supporting information

S1 Checklist COREQ-32-item checklist. (XLSX)

S1 Fig Map of Kenya showing Isiolo County (Map developed by the corresponding author using QGIS version 2.16.3 with geographical data from https://africaopendata.org/dataset/kenya-counties-shapefile). (PDF)

S1 Questionnaire Community KAP Questionnaire. (DOC)

S1 Dataset Quantitative data set. (XLSX)

S1 File FGD study guide. (DOC)
