# Supplementary figures and images for: Knowledge, attitude and practices on cholera in an arid county, Kenya, 2018: A mixed-methods approach
Source: PLoS One. 2020 Feb 26;15(2):e0229437. doi: 10.1371/journal.pone.0229437 (PMC7043758; doi:10.1371/journal.pone.0229437)

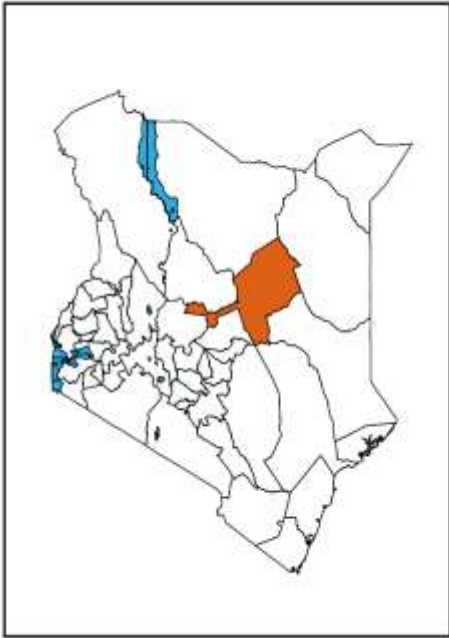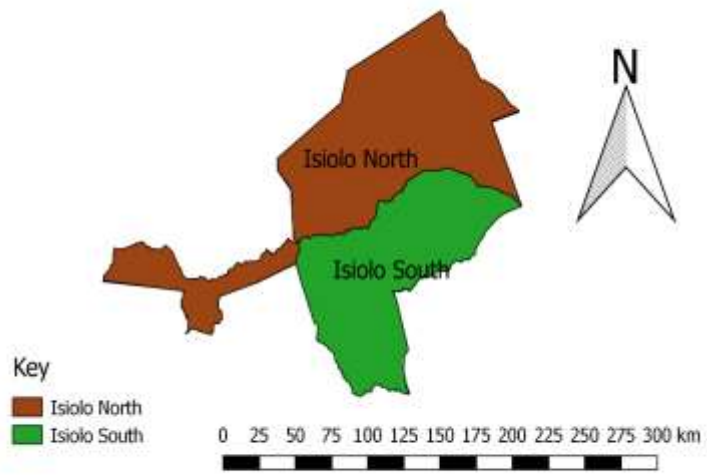

Supplement: S1 Fig — (PDF) [file pone.0229437.s002.pdf]
