# Supplementary material for: Knowledge, attitude and practices on cholera in an arid county, Kenya, 2018: A mixed-methods approach
Source: PLoS One. 2020 Feb 26;15(2):e0229437. doi: 10.1371/journal.pone.0229437 (PMC7043758; doi:10.1371/journal.pone.0229437)
Supplement: S1 Questionnaire — (DOC) [file pone.0229437.s003.doc]

**Date of Interview__________ Interviewer_____________ Community Health Volunteer____________**

**County_______________Sub-county________________Village/Town___________________**

**GPS Coordinates of Household: Longitude____________________ Latitude____________________**

**Hello, my name is _______________. I am working with the Kenyan Ministry of Health to investigate illnesses in the community. We have a few questions about illness in the community and water issues. This may take about 30-40 minutes. May I please speak to the person in the home who usually takes care of the ill family members and brings the water for the family?**

***If YES, begin the interview. If NO, thank you*** *(If possible, try to record demographics for refusals)*

| 1. What is your age in years? | 1. Gender | 1  0 | Male  Female |
| --- | --- | --- | --- |
|  |

**Background Socioeconomic & Education**

| 1. How many people live in your household? |  |  |
| --- | --- | --- |
| 1. How many children less than 5 years old live in your household? |  |  |

**Cholera General Knowledge Information**

| 1. Have you ever heard of an illness called cholera? | 1  0  99 | Yes  No  Don’t Know |
| --- | --- | --- |
| 1. Have you heard about the cholera outbreak in your area recently? | 1  0  99 | Yes  No  Don’t Know |
| 1. Can you tell me what the main symptoms of cholera are?   ***(Do not read. Check all that are mentioned.)*** | 1  2  3  4  5  6  7  99 | Watery diarrhea  Bloody diarrhea  Vomiting  Fever  Dehydration  Decreased appetite  Other(specify)____________________  Don’t Know |
| 1. Do you know what causes cholera?   ***(Do not read. Check all that are mentioned.)*** | 1  2  3  4  5  6  7  8  99 | Drinking bad water  Eating bad food  Unwashed fruit/vegetables  Flies/Insects  Poor hygiene  Open defecation  Spirits/Curse/Bad Omen  Other (specify) ____________________  Don’t Know |
| 1. Can cholera spread from one person to another? | 1  0  99 | Yes  No  Don’t know |
| 1. How severe is cholera compared to other types of diarrhea? ***(Read all choices. Choose only 1.)*** | 1  2  3  99 | Less severe  Equal severity  More severe  Don’t know |
| 1. How can you prevent you or your family members from getting cholera?   ***(Do not read. Check all that are mentioned. Prompt after each response.)*** | 0  1  2  3  4  5  6  7  8  9  10  99 | Cannot prevent  Herbs  Wash hands  Cook food thoroughly  Reheat stored food  Cover food  Boil or treat water  Wash vegetables and fruit  Clean cooking utensils/vessels  Use a latrine/Avoid open defecation  Other (specify)____________________  Don’t Know |
| 1. Where would you go for care if you or your family member had cholera?   ***(Do not read. Check all that are mentioned. Prompt after each response)*** | 1  2  3  4  5  6  7  8  9  99 | Private clinic  Hospital/ Government facility  Dispensary/ health center  Community health worker  Chemist  Kiosk/shop  Traditional healer  Family or neighbor  Other (specify)_____________________  Don’t know |
| 1. How can you treat cholera for yourself or your family members when you are at home and cannot get to a health facility?   ***(Do not read. Check all that are mentioned. Prompt after each response)*** | 1  2  3  4  5  6  7  8  9  10  11  12  13  99 | Increase liquid intake  Decrease liquid intake  Increase food intake  Decrease food intake  Use oral rehydration solution (ORS)  Use sugar-salt solution  Pill or syrup medicine  Injection  Go to church/ mosque/religious place  Go to traditional healer  Home remedy (specify)______________  Other (specify)_____________________  Do not treat  Don’t Know |

**Cholera in Your Village/Neighborhood**

| 1. Have you heard that people in your village/neighborhood had cholera in the past 6 months? | 1  0  99 | Yes 🡪 **Go to 15**  No **🡪 Go to 20**  Don’t Know **🡪 Go to 20** |
| --- | --- | --- |
| 1. When was the most recent time you heard of cholera in your village/neighborhood? | 0  1  2  3  4  99 | Never heard  Past 7 days  In the past month  Between 2-6 months  Over 6 months ago  Don’t know |
| 1. Have you heard that people in your village/neighborhood died from cholera in the past 6 months? | 1  0  99 | Yes  No  Don’t know |
| 1. Please tell me all the ways you heard about the cholera outbreak.   ***(Do not read. Check all that are mentioned. Prompt after each response.)*** | 1  2  3  4  5  6  7  8  9  10  11  12  13  14  15  99 | Family member  Neighbor  Friend  Chief (*Baraza*) Community Meeting  Community health worker/ volunteer  Health Worker  Women’s group  Church, Mosque or religious group  School  NGO or Volunteer Organization (ex. Red Cross, MSF, UNICEF, ACF)  Radio  Electronic media (TV, internet, facebook)  Newspaper  Poster or Wall Hanging  Other (specify)__________  Don’t know |
| 1. Did you hear messages about how to prevent cholera from these sources of information?   ***(Please refer to sources identified in question 4.)*** | 1  0  99 | Yes **🡪 Go to 19**  No **🡪 Go to 20**  Don’t know **🡪 Go to 20** |
| 1. What did you hear?   ***(Do not read. Check all that are mentioned. Prompt after response.)*** | 1  2  3  4  5  6  7  8  9 | Boil or treat water  Build/Use latrines/Avoid open defecation  Wash hands  Cover food  Cook food thoroughly  Wash vegetables and fruit  Clean cooking utensils/vessels  Seek treatment if you have severe, watery bloody diarrhea  Other________________________ |

**Cholera in Family Member**

| 1. Did you or any of your family members become ill with cholera in the past 6 months? | 1  0  99 | Yes **🡪 Go to 21**  No **🡪 Go to 38**  Don’t know **🡪 Go to 38** |
| --- | --- | --- |
| 1. How many family members became ill with cholera? |  |  |
| 1. Have there been any deaths in your family due to cholera in the past 6 months? | 1  0 | Yes **🡪 Go to 23**  No **🡪 Go to 24** |
| 1. How many family members passed away due to cholera? |  |  |

| 1. Did you use any of the following to treat yourself or your family member in the home when having diarrhea? ***(Ask each item. Choose Yes, No or Don’t know for each item)*** | | | |
| --- | --- | --- | --- |
| 1. Herbal Treatment | Yes (1) | No (0) | Don’t Know (99) |
| 1. Fluid prepared from ORS packet | Yes (1) | No (0) | Don’t Know (99) |
| 1. Other solution prepared at home | Yes (1) | No (0) | Don’t Know (99) |
| 1. Oral medicine/Antibiotics | Yes (1) | No (0) | Don’t Know (99) |
| 1. Other (specify)______________________ | Yes (1) | No (0) | Don’t Know (99) |

| 1. Did you or your family member seek care for cholera? | 1  0  9 | Yes **🡪 Go to 26**  No **🡪 Go to 36**  Don’t know **🡪 Go to 36** |
| --- | --- | --- |
| 1. When was the last time you sought care for cholera for you or your family member? | 1  2  3  4  99 | In past 7 days  Between 1 week - 1 month ago  Between 1 month - 6 months ago  Over 6 months ago  Don’t know |
| 1. Who was the person you last sought care for cholera? | 1  2  3 | Respondent  Respondent’s family member  🡪 Age of family member_______years  Other (specify)_______________  🡪 Age of other person _________years |

***(The following questions 28 – 37 are about the person identified in question 27)***

| 1. Did you/your family member seek care at: | | | |
| --- | --- | --- | --- |
| 1. Hospital/Government Facility | Yes (1) | No (0) | Don’t Know (99) |
| 1. Cholera Treatment Center | Yes (1) | No (0) | Don’t Know (99) |
| 1. Private Clinic | Yes (1) | No (0) | Don’t Know (99) |
| 1. Dispensary/Health Center | Yes (1) | No (0) | Don’t Know (99) |
| 1. Chemist | Yes (1) | No (0) | Don’t Know (99) |
| 1. Kiosk/Shop | Yes (1) | No (0) | Don’t Know (99) |
| 1. Community Health Worker | Yes (1) | No (0) | Don’t Know (99) |
| 1. Traditional Healer | Yes (1) | No (0) | Don’t Know (99) |
| 1. Spiritual Leader | Yes (1) | No (0) | Don’t Know (99) |
| 1. Other (specify)___________________________ | Yes (1) | No (0) | Don’t Know (99) |

Health Facility=Government Hospital, Cholera Treatment Center Private Clinic, Dispensary

**If YES to Health Facility, 🡪 Go to 29**

**If NO or Don’t know to Health Facility  Go to 38**

| 1. What did they give you at the health facility to treat your cholera illness? ***(Read all choices and check all that apply.)*** | 1  2  3  4  5  6  7  8  9  99 | ORS  Fluid through a needle / IV Fluids  Syrup or pill medicine by mouth  Injection  Antibiotics  Anti-motility medicine  Zinc sulfate  Special air through a mask / Oxygen  Other (specify)_________________  Don’t Know |
| --- | --- | --- |
| 1. Were you/your family member hospitalized overnight? | 1  0  99 | Yes  No  Don’t know |
| 1. Did the hospital take a blood test from you/your family member? | 1  0  99 | Yes  No  Don’t know |
| 1. Did the hospital take a stool test from you/your family member? | 1  0  99 | Yes  No  Don’t know |
| 1. What did the doctor/hospital give you/your family member to take home to treat cholera? | 0  1  2  3  4 | Nothing  ORS Packet(s)  Syrup or Pill  Antibiotic Medicine  Anti-motility Medicine |
| 1. Did anyone at the health facility talk to you about preventing cholera? | 1  0  99 | Yes **🡪 Go to 35**  No **🡪 Go to 36**  Don’t know **🡪 Go to 36** |
| 1. What did they talk about? ***(Do not read. Check all mentioned. Prompt after response).*** | 1  2  3  4  5  6  7  8  9  10  11  99 | Treat water  Build and use latrines  Wash hands  Cover food  Cook food thoroughly  Reheat stored food  Wash vegetables and fruit  Clean cooking utensils/ vessels  Seek treatment if severe, watery, bloody diarrhea  Diarrhea and children  Other (specify) _______________  Don’t know |
| 1. How long does it take to get to the health facility from your home? | #  99 | _____ minutes  _______ hours  ______days  Don’t know |
| 1. How difficult is it to get to the health facility?   ***(Read responses and check all that apply.)*** | 1  2  3  99 | Not difficult  Somewhat difficult  Very difficult  Don’t Know |

Oral Rehydration Solution (ORS)

| 1. Has anyone taught you how to prepare a home-made rehydration solution at home to treat diarrhea? | 1  0  99 | Yes **🡪 Go to 39**  No **🡪 Go to 41**  Don’t know **🡪 Go to 41** |
| --- | --- | --- |
| 1. Who taught you to prepare the solution?   ***(Do not read. Check all mentioned.)*** | 1  2  3  4  5  6  7  8  9  10  11  12  13  14  15  99 | Spouse  Mother  Mother-in-law  Father  Father-in-law  Co-wife  Government Hospital/Clinic  Private clinic  Community health worker/ volunteer  Traditional healer  Spiritual healer  Village chief  Older woman in community  Older man in community  Other (specify)________________  Don’t know |
| 1. What does this solution contain?   ***(Do not read. Check all mentioned.)*** | 1  2  3  4  5  6  7  8  99 | Sugar  Salt  Herbs  Water  Tea  Other fluid (specify)_______________  Contents of ORS Packet  Other (specify)_________________  Don’t know |
| 1. Have you heard of Oral Rehydration Solution or ORS? | 1  0  99 | Yes **🡪 Go to 42**  No **🡪Go to 52**  Don’t know **🡪 Go to 52** |
| 1. From who or where have heard of ORS?   ***(Do not read. Check all that are mentioned.)*** | 1  2  3  4  5  6  7  8  9  10  11  12  13  14  15  99 | Family member  Neighbor  Friend  Chief (Baraza) Community Meeting  Community health worker/ volunteer  Health worker  Women’s group  NGO or Volunteer Organization (ex. Red Cross, MSF, UNICEF. ACF)  Radio  Electronic media such as TV, internet  Newspaper  Poster or wall hanging  School  Health Facility  Other, Specify _______________  Don’t know |
| 1. What is ORS used to treat? | 1  2  3  4  99 | Dehydration  Diarrhea  Children’s illnesses  Other (specify)___________________  Don’t Know |
| 1. Do you know how to prepare ORS? | 1  0  99 | Yes  No  Don’t know |
| 1. Is ORS available in your village? | 1  0  99 | Yes  No  Don’t know |
| 1. Where is it available?   ***(Do not read. Check all that are mentioned.)*** | 1  2  3  4  5  6  99 | Health care facility  Chemist/Pharmacy  Kiosk/Shop in Village  Supermarket  NGO  Other (specify)__________________  Don’t know |
| 1. How much does one ORS packet cost? | 1  2  99 | ____________Ksh **🡪 Go to 48**  Can get it free at health facility **🡪 Go to 50**  Don’t know **🡪 Go to 50** |
| 1. How do you find the price of ORS?   ***(Read all choices. Mark only 1)*** | 1  2  3 | Cheap  Fair  Expensive |
| 1. Have you purchased ORS in the past 6 months? | 1  0  99 | Yes  No  Don’t know |
| 1. Do you have one or more packets of ORS in the home? | 1  0  99 | Yes **🡪 Go to 51**  No **🡪 Go to 52**  Don’t know **🡪 Go to 52** |
| 1. May I see the ORS packet(s)? | 1  0  99 | Present  Absent  Refused |

**Water and Water Treatment Information**

| 1. What is the main source of your household’s drinking water during the DRY season? ***(Do not read; Choose 1)*** | 1  2  3  4  5  6  7  8  9  10  11  12  13  14 | Open deep well  Protected deep well  Shallow well/hand-dug well  Spring  Lake  Pond/Seasonal lake  River  Borehole  Rain water catchment from roof  Piped water to house  Community tap  Water vendor  Dam  Other (specify)________________ |
| --- | --- | --- |
| 1. What is your main source of drinking water during the RAINY season? ***(Do not read; Choose 1)*** | 1  2  3  4  5  6  7  8  9  10  11  12  13  14 | Open deep well  Protected deep well  Shallow well/hand-dug well  Spring  Lake  Pond/Seasonal lake  River  Borehole  Rain water catchment from roof  Piped water to house  Community tap  Water vendor  Dam  Other (specify)________________ |
| 1. Where are you presently getting your water?   ***(Do not read; Choose 1)*** | 1  2  3  4  5  6  7  8  9  10  11  12  13  14 | Open deep well  Protected deep well  Shallow well/hand-dug well  Spring  Lake  Pond/Seasonal lake  River  Borehole  Rain water catchment from roof  Piped water to house  Community tap  Water vendor  Dam  Other (specify)________________ |
| 1. Are there any times during the year, when water is not readily available? | 1  0 | Yes **🡪 Go to 56**  No **🡪 Go to 58** |
| 1. During the past 6 months, how often was water not readily available? ***(Read choices. Choose only 1.)*** | 1  2  3  4  6  99 | One week during past 6 months  One month during past 6 months  1- 3 months during past 6 months  Over 3 months during past 6 months  Other (specify)_______________  Don’t know |
| 1. Why was water not readily available? | 1  2  3  4  99 | Drought  Water rationing  Broken pipes/ water system  Other (specify)_________________  Don’t know |
| 1. Do you do something to your drinking water to make it safe to drink? | 1  0  99 | Yes **🡪** **Go to 59**  No **🡪 Go to 60**  Don’t know **🡪 Go to 60** |
| 1. What do you do to treat the water?   ***(Do not read. Check all that are mentioned. Prompt after each response.)*** | 1  2  3  4  5  6  7  8  9  10  11  12  13  99 | Boil  Decanting  Keep water in hot sun  Filter  Cloth filter  Sand (shallow dug well)  Alum  WaterGuard  PuR  AquaGuard  Aquatabs  Use a ceramic/biosand filter  Other *(Specify*)_________________  Don’t know |
| 1. Have you ever heard about water treatment products? | 1  0  99 | Yes **🡪 Go to 61**  No **🡪 Go to 66**  Don’t know **🡪Go to 66** |
| 1. Which water treatment product have you heard of? | 1  2  3  4  5 | WaterGuard  PuR  AquaGuard  Aquatabs  Other (specify)______________ |
| 1. In the last 6 months, have you ever received any water treatment products or hygiene products for free from the government, NGO, or another organization? | 1  0  99 | Yes **🡪 Go to 63**  No **🡪 Go to 68**  Don’t know **🡪Go to 68** |
| 1. What were you given?   ***(Do not read. Check all that are mentioned.)*** | 1  2  3  4  5  6  7  8  9  10  11  12  13  14  15  16 | WaterGuard **🡪 Go to 64**  PuR **🡪 Go to 64**  AquaGuard **🡪 Go to 64**  Aquatabs/chlorine tabs **🡪 Go to 64**  Bottles of chlorine **🡪 Go to 64**  Drums of chlorine **🡪 Go to 64**  Soap  Jerrycan  Bucket  Ceramic water filter  **Go to 68**  Medicine/Antibiotics  ORS  Print material  Incentives  Advice  Other____________________ |
| 1. Were you given any counseling or education on how to use these water treatment products? | 1  0  99 | Yes  No  Don’t know |
| 1. Did you use any of these products? | 1  0  99 | Yes **🡪 Go to 66**  No 🡪 **Go to 67**  Don’t know 🡪 **Go to 68** |
| 1. What did you use?   **🡪 Go to 68** | 1  2  3  4  5  6  99 | WaterGuard  PuR  AquaGuard  Aquatabs/chlorine tabs  Bottles of chlorine  Drums of chlorine  Don’t know |
| 1. Why did you not use these products? | 1  2  3  4  5  6  99 | Bad Taste  Dangerous to use these products  No container to treat water  No need to treat water  Did not know how to use the product  Other (specify)_________________  Don’t know |

**Handwashing Information**

| 1. When do you wash your hands?   ***(Do not read. Check all that are mentioned.)*** | 1  2  3  4  5  6  7  8  99 | After using the toilet  Before eating  After eating  When serving meals  Before cooking  After cleaning babies when they defecate  Other (Specify) ___________________  Never wash hands  Don’t Know |
| --- | --- | --- |
| 1. Do you have soap in the house? | 1  0  99 | Yes  No  Don’t know |
| 1. For which purposes, do you use the soap?   ***(Do not read. Check all that are mentioned).*** | 1  2  3  4  5  99 | Washing hands  Laundry  Cleaning utensils/ vessels  Bathing  Other (*Specify)*______________________  Don’t know |

**Education/Socioeconomic/Personal** Information

| 1. Can you read and write? | 1  0  99 | Yes  No  Don’t know |
| --- | --- | --- |
| 1. What is the highest level of education you have attended? ***(Choose only 1)*** | 0  1  2  3  4  99 | None  Lower Primary  Upper Primary  Secondary or Higher  Other (specify)______  Don’t know |
| 1. Does your household have the following?   ***(Read all choices. Mark all that apply.)*** | 1  2  3  4  5  6  7  8  9  10  0 | Electricity  Television  Radio  Animal-drawn cart  Motorcycle/Scooter  Bicycle  Car/truck  Refrigerator  Telephone (mobile or non-mobile)  Agricultural land  None of the above |
| 1. What type of cooking fuel does your household use? ***(Read all choices. Mark all that apply.)*** | 1  2  3  4  5  6  7  8  9  10  0 | Charcoal  Wood  Straw/shrubs/grass  Animal dung  Agricultural crop residue  Electricity  Liquid Propane Gas  Natural Gas  Kerosene  Other (specify)____________________  None |
| 1. What is the main source of family income?   ***(Do not read. Choose only 1.)*** | 1  2  3  4  5  6  99 | Small Business/Trader  Fishing  Farmer  Employed/Salaried  Unskilled labor  Unemployed  Don’t Know |
| 1. What is your level of education?   ***(Do not read. Check all that are mentioned.)*** | 1  2  3  4  5 | None  Primary complete  Primary complete  Secondary or higher  Don’t know |

**Home Information/Observations**

| 1. Where do you defecate? May I see your latrine?   **(*Mark what is seen.* *Do not read. Circle the one that applies.)*** | 1  2  3  4  5  6  7 | Flush Latrine  Covered pit latrine  Uncovered dry pit latrine  Flying toilet  Bush  Lake or River  Other, (Specify) _____________________ |
| --- | --- | --- |
| 1. What is the main roofing material for the household’s dwelling? ***(Choose 1.)*** | 1  2  3  4  5  6  7 | Thatch  Metal/Iron Sheets  Tile/Asbestos sheets  Wood  Cement  None; no household dwelling/structure  Others (Specify)______________________ |
| 1. What is the main flooring material? ***(Choose 1)*** | 1  2  3  4  5  6  7  8  9 | Dung  Earth/ sand/ mud  Metal  Wood  Broken bricks  Cement  Tile  None; no household dwelling/structure  Other (Specify) ______________________ |
| 1. What is the material used for the walls?   ***(Choose 1)*** | 1  2  3  4  5  6  7  8 | Dung/Mud  Metal sheets  Twigs  Wood  Cement/Plaster  Bricks/blocks/stones  None; no household dwelling/structure  Other (Specify) _______________________ |
| 1. May I see where you store your water?   ***(Mark all that are seen.)*** | 1  2  3  4  5  6 | Jerrycan  Bucket  Pot  Cooking pot *(Sufuria)*  Refused  None |
| 1. May I see the products you have purchased or have received from the government or NGOs?   ***(Mark all that are seen.)*** | 1  2  3  4  5  6  7  8  9  10  11  12  13 | Soap  WaterGuard  PuR  Aquatabs/chlorine tabs  Bottles of chlorine  Drums of chlorine  Ceramic water filter  Medicine/Antibiotics  ORS  Food  Print material  Other (specify)_______________________  None in the home |
| 1. May I test a sample of drinking water to see if there is chlorine in it? | 1  2  3  4  5 | Chlorine test performed **🡪 Go to 84**  No water stored  Refused  Test not done  Other (specify)______________________ |
| 1. Free chlorine residuals measured by Hach Pocket Colorimeter II |  | _._ _ mg/L (#, 2 decimal places) |

**“The interview is now finished. Thank you for your time.”**
