# Supplementary material for: Knowledge, attitude and practices on cholera in an arid county, Kenya, 2018: A mixed-methods approach
Source: PLoS One. 2020 Feb 26;15(2):e0229437. doi: 10.1371/journal.pone.0229437 (PMC7043758; doi:10.1371/journal.pone.0229437)
Supplement: S1 File — (DOC) [file pone.0229437.s005.doc]

**Assessing Knowledge, attitude and practices towards cholera in Isiolo County**

**FOCUS GROUP: DISCUSSION GUIDE**

**Facilitator’s welcome, introduction and instructions to participants (15 minutes)**

**Welcome** and thank you for volunteering to take part in this focus group. You have been asked to participate as your point of view is important. I realize you are busy and I appreciate your time.

**Introduction:** This focus group discussion is designed to assess your current knowledge, attitude and practices that contribute to prevention of cholera and other diarrheal diseases. The focus group discussion will take no more than two hours. I also request to tape the discussion so as to facilitate its recollection? (If yes, switch on the recorder)

**Anonymity:** Despite being taped, I would like to assure you that the discussion will be anonymous. The tapes will be kept safely in a locked facility until they are transcribed word for word, then they will be destroyed. The transcribed notes of the focus group will contain no information that would allow individual subjects to be linked to specific statements. You should try to answer and comment as accurately and truthfully as possible. I and the other focus group participants would appreciate it if you would refrain from discussing the comments of other group members outside the focus group. If there are any questions or discussions that you do not wish to answer or participate in, you do not have to do so; however please try to answer and be as involved as possible.

**Ground rules**

- The most important rule is that only one person speaks at a time. There may be a temptation to jump in when someone is talking but please wait until they have finished.
- There are no right or wrong answers
- You do not have to speak in any particular order
- When you do have something to say, please do so. There are many of you in the group and it is important that I obtain the views of each of you
- You do not have to agree with the views of other people in the group
- Does anyone have any questions? (Answer any questions from the participants).
- OK, let’s begin

**Warm up**

- First, I’d like everyone to introduce themselves. Please tell us your name ?

**Introductory question**

I am going to give you a few minutes to think about your experience with environmental hygiene, water and sanitation in your wards. Is anyone happy to share his or her experience?

**Guiding questions**

**Knowledge (20 minutes discussion)**

- What do you think causes cholera?
- How would you know that a person is infected with cholera?
- How can cholera be prevented?
- What is the treatment for cholera?

**Attitude (20 minutes discussion)**

- What do you think could be the reason behind increase in diarrheal diseases in your ward?
- Are you interested in preventing cholera in your area? (If no, why?)
- How does cholera compare with other diarrheal illnesses in terms of severity?
- What do you think about water treatment products?(availability and use)

**Practices (20 minutes discussion)**

- What is your source of drinking water?
- Where do you defecate? Do you use communal pit latrines?
- When do you wash your hands?
- How do you treat your drinking water?

**Concluding question**

- Of all the issues we have discussed today, what would you say are the most important issues you would like to express regarding cholera/diarrheal disease prevention?

**Conclusion**

- Thank you for participating. This has been a very successful discussion
- Your opinions will be a valuable asset to the study
- We hope you have found the discussion interesting
- I would like to remind you that any comments featuring in this report will be anonymous

Please, write your report based on the results of the focus group. Please remember to maintain confidentiality of the participating individuals by not disclosing their names.
